# In Silico Study of Polyunsaturated Fatty Acids as Potential SARS-CoV-2 Spike Protein Closed Conformation Stabilizers: Epidemiological and Computational Approaches

**DOI:** 10.3390/molecules26030711

**Published:** 2021-01-29

**Authors:** Alonso Vivar-Sierra, María José Araiza-Macías, José Patricio Hernández-Contreras, Arely Vergara-Castañeda, Gabriela Ramírez-Vélez, Rodolfo Pinto-Almazán, Juan Rodrigo Salazar, Marco A. Loza-Mejía

**Affiliations:** 1 Design, Isolation, and Synthesis of Bioactive Molecules Research Group, Chemical Sciences School, Universidad La Salle-México, Benjamín Franklin 45, Mexico City 06140, Mexico; alonso.vivar@lasallistas.org.mx (A.V.-S.); mj.araiza@lasallistas.org.mx (M.J.A.-M.); jp.hernandez@lasallistas.org.mx (J.P.H.-C.); gabriela.ramirez@lasalle.mx (G.R.-V.); juan.salazar@lasalle.mx (J.R.S.); 2 Basic and Clinical Health Sciences Research Group, Chemical Sciences School, Universidad La Salle-México, Benjamín Franklin 45, Mexico City 06140, Mexico; arely.vergara@lasalle.mx; 3 Molecular Biology in Metabolic and Neurodegenerative Diseases Laboratory, Research Unit, High Speciality Regional Hospital of Ixtapaluca (HRAEI), Carretera Federal México-Puebla Km 34.5, Ixtapaluca 56530, Mexico; rodolfopintoalmazan@gmail.com

**Keywords:** COVID-19, SARS-CoV-2, PUFA, molecular docking, spike protein, DHA

## Abstract

SARS-CoV-2 infects host cells by interacting its spike protein with surface angiotensin-converting enzyme 2 (ACE2) receptors, expressed in lung and other cell types. Although several risk factors could explain why some countries have lower incidence and fatality rates than others, environmental factors such as diet should be considered. It has been described that countries with high polyunsaturated fatty acid (PUFA) intake have a lower number of COVID-19 victims and a higher rate of recovery from the disease. Moreover, it was found that linoleic acid, an omega-6 PUFA, could stabilize the spike protein in a closed conformation, blocking its interaction with ACE2. These facts prompted us to perform in silico simulations to determine if other PUFA could also stabilize the closed conformation of spike protein and potentially lead to a reduction in SARS-CoV-2 infection. We found that: (a) countries whose source of omega-3 is from marine origin have lower fatality rates; and (b) like linoleic acid, omega-3 PUFA could also bind to the closed conformation of spike protein and therefore, could help reduce COVID-19 complications by reducing viral entrance to cells, in addition to their known anti-inflammatory effects.

## 1. Introduction

Coronaviruses are a diverse group of viruses, characterized by crown-shaped spikes on their surface and have a single-stranded RNA as their genetic material. Seven types of coronaviruses can infect humans; four (HCoV-229E, HCoV-OC43, HCoV-NL63, and HCoV-HKU1) cause mild infections that only affect the upper respiratory tract. Meanwhile, the other three (SARS-CoV, MERS-CoV, and SARS-CoV-2) cause severe respiratory diseases [1,2]. In December 2019, a new virus belonging to the coronavirus family (SARS-CoV-2) was identified in Wuhan, Hubei province, China, [3] and soon spread worldwide, causing the World Health Organization (WHO) to declare it as a pandemic in March 2020 [4]. Despite the efforts invested by governments, health services, and research groups, worldwide 62,844,837 cases have been identified, and it has caused 1,465,144 deaths to date (1 December 2020).

SARS-CoV-2 is spread mainly from person to person through droplets released from an infected person’s nose or mouth when they cough, sneeze, or speak [5,6]. This disease’s symptoms can vary, but include a fever, runny nose, cough, difficulty breathing, tiredness, and loss of the sense of taste and smell. However, some patients do not have any symptoms, which has made this disease easier to spread [4,7]. The virus that causes COVID-19 disease is composed of approximately 30,000 nucleotides that make up its genome, which encodes for four structural proteins: membrane protein (M), nucleocapsid protein (N), envelope protein (E), spike protein (S), as well as other nonstructural proteins [4].

**Figure 1:**
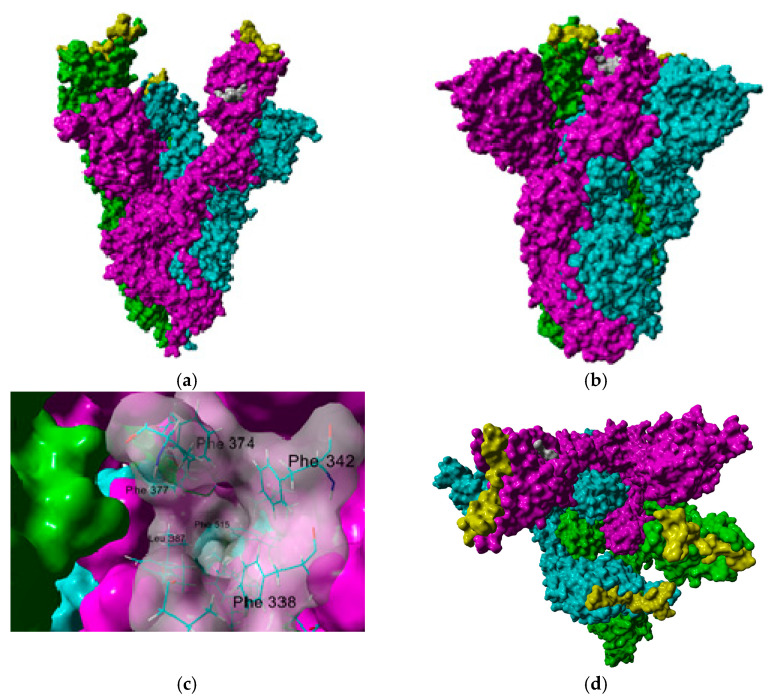

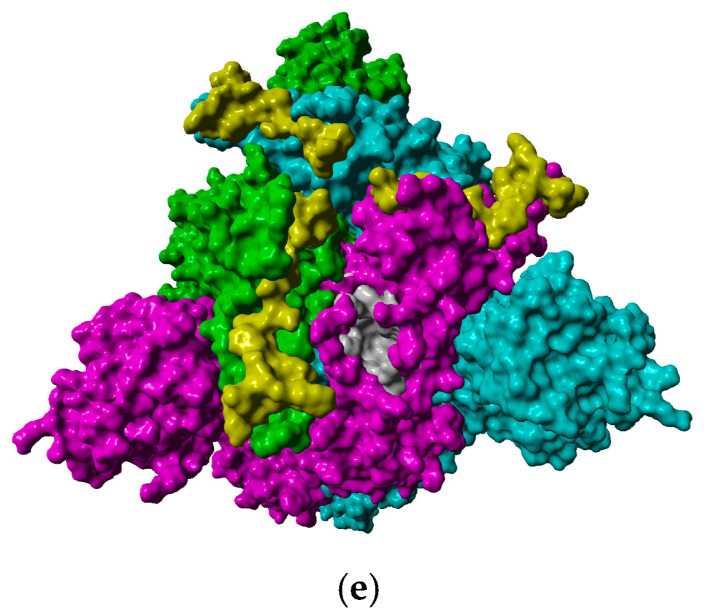
Lateral view of spike protein in its (**a**) open and (**b**) closed conformations. Subunits A, B and C are colored in cyan, green, and magenta, respectively. The fatty acid-binding pocket is colored in white, and the site for angiotensin-converting enzyme (ACE) binding is colored in yellow. A closer view of the fatty acid binding pocket, with most relevant residues labeled, is shown in (**c**). Upper views of both open (**d**) and closed (**e**) conformations are also shown.

SARS-CoV-2 attaches to a host cell when the spike protein trimer (S) binds to the receptor angiotensin-converting enzyme 2 (ACE2) [2,3,8]. Thus, S-protein has become an interesting target for the development of vaccines and drug treatments. Many S-protein structures have become available, revealing some interesting features such as its high flexibility, especially in the receptor-binding domain (RBD). This domain exhibits two different conformations: the up-conformation, which is “open” for receptor-binding; and a down-conformation, which is “closed” and inaccessible for receptor-binding [9,10]. On the other hand, a cryo-EM structure of SARS-CoV-2 spike (S) glycoprotein revealed that the receptor-binding domain also binds to linoleic acid (LA), an essential free fatty acid, in three composite binding pockets called the fatty acid-binding pockets [11]. It was identified that when LA binds to the RBD, it displays a closed conformation that is expected to reduce ACE2 binding (Figure 1). This fact raises two critical questions. The first being: could the fatty acid-binding pocket be used for the development of new drugs? This strategy had been previously explored for the development of antivirals for the treatment of rhinovirus infections [12,13,14]. The second question: could the levels of LA or polyunsaturated fatty acids (PUFA) be related to the development of severe symptoms in COVID-19 patients? Therefore, could its supplementation be helpful in the treatment of the disease? [15].

PUFA, including omega 3 (ω-3 PUFA) and omega 6 (ω-6 PUFA), constitute a nutrient family of great importance for human beings. PUFAs are considered essential because they are needed in the diet at adequate proportions; our bodies cannot introduce unsaturations in these positions. The most important ω-3 PUFAs are α-linolenic acid (ALA), eicosapentaenoic acid (EPA), and docosahexaenoic acid (DHA), while ω-6 PUFA mainly includes linoleic acid (LA) and arachidonic acid (AA) [16,17]. Interest in ω-3 PUFA’s role in health promotion and disease risk reduction has increased in the last years. It has been suggested that a diet low in ω-3 PUFA consumption (average daily consumption less than 430–470 mg of EPA and DHA) is the twelfth leading dietary risk factor for attributable disability-adjusted life years (DALYs) (7.41 million) and is responsible for nearly 337,000 deaths [18,19]. According to the Nutrition and Chronic Diseases Expert Group (NutriCoDE) as part of the 2010 Global Burden of Diseases, Injuries, and Risk Factors (GBD) Study, the global average intake of seafood ω-3 fats was 163 mg/day, with tremendous regional variation (from <50 to >700 mg/day) and national variation (5–3886 mg/day). The dietary intake of EPA and DHA per day varies, according to ref 18. However, the most common recommendation is to include at least two servings of fish per week, providing a minimum average of nearly 300–450 mg/day. Interestingly, individuals from countries with high ω-3 PUFA intake show less risk of inflammatory diseases, such as Crohn’s disease and ulcerative colitis. Additionally, COVID-19 data from these countries show a lower number of victims and fewer inflammatory complications, with a higher recovery rate from the disease [20].

**Figure 2:**
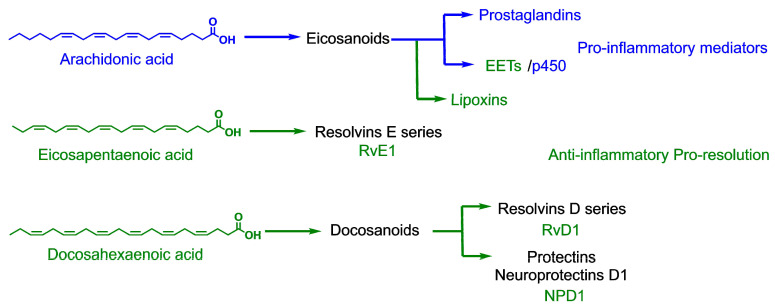
Metabolic pathways of polyunsaturated fatty acid (PUFA) that lead to pro-inflammatory and anti-inflammatory mediators. EET: Epoxyeicosatrienoic acids.

The potential beneficial role of PUFAs in COVID-19 has been explained from their role in inflammation, because they are substrates for the synthesis of lipid mediators, such as eicosanoids [20,21]. Besides normal immune responses, SARS-CoV-2 initiates the process of inflammation in the host cells, activating the production of pro-inflammatory cytokines such as tumor necrosis factor (TNF-α), interleukin 6 (IL-6), and interleukin 1 beta (IL-1β). Additionally, proteins released by infected pneumocytes known as damage-associated molecular patterns (DAMPs) likely combine with viral pathogen-associated molecular patterns (PAMPs) to activate innate immunity [22]. This process leads to the activation of phospholipase A2, which cleaves arachidonic acid (AA). Upon downstream modification by cyclooxygenases or lipoxygenases, AA produces pro-inflammatory eicosanoids such as prostaglandins, prostacyclins, thromboxanes, and leukotrienes (Figure 2). The effects of these mediators and the increased production of pro-inflammatory chemoattractants such as VEGF and MCP-1 usually cause vasodilation, allowing immune cells such as monocytes and neutrophils to leak from the bloodstream into the tissue, where they become activated and release the enzymatic contents of their granules to the environment in an unspecific manner, amplifying the damage to the tissue and the inflammatory process [23].

On the other hand, the eicosanoids derived from EPA and DHA are less inflammatory than those derived from AA. If the proportion of EPA and DHA exceeds that of AA in the cell’s membrane phospholipids, there will be a reduced synthesis of AA-lipid mediators. EPA and DHA also inhibit leukocyte chemotaxis, reduce adhesion molecule expression and leukocyte-endothelial adhesive interactions, and inhibit the activation of the nuclear factor NF-κB (nuclear factor kappa-light-chain-enhancer of activated B cells), with the consequent decrease in pro-inflammatory cytokines [24]. Furthermore, it has been shown that the enzymatic oxidation of EPA and DHA leads to the synthesis of specialized pro-resolving mediators (SPMs), such as resolvins, protectins, and maresins, and the synthesis of fewer inflammatory eicosanoids, which reduce inflammation (Figure 2). Resolvins produced from EPA and DHA, and protectins produced from DHA, are involved in the COX and LOX pathways and are inflammation-resolving, inhibiting the transendothelial migration of neutrophils and cytokines (IL-1β and TNF-α) and chemokine production [24].

**Figure 3:**
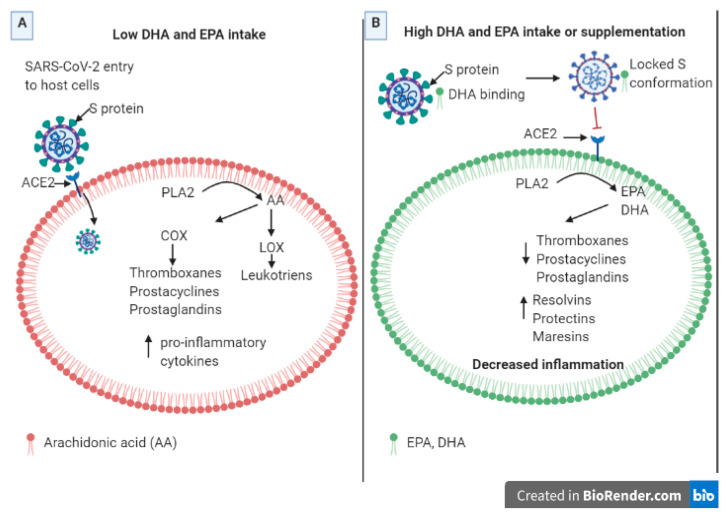
Potential role of docosahexaenoic acid (DHA), eicosapentaenoic acid (EPA), and other ω-3 PUFA in reducing COVID-19 infection and complications. (**A**) With a low intake of omega-3 fatty acids, such as EPA and DHA, SARS-CoV-2 mediates infection to host cells through an interaction of S-protein and ACE2. Membranes of immune cells would have a higher proportion of arachidonic acid (AA), instead of EPA and DHA, leading to the production of inflammatory eicosanoids and pro-inflammatory cytokines. (**B**) With a high dietary intake or supplementation of EPA and DHA, these omega-3 fatty acids could bind to the S-protein of SARS-CoV-2, locking it in an inactive conformation and preventing the interaction of S-protein with ACE2 and, therefore, the infection of the virus. Membranes of immune cells have a higher proportion of omega-3 acids rather than omega-6 acids, activating the production of inflammation resolving mediators, such as resolvins, protectins, and maresins, and producing fewer pro-inflammatory cytokines. These would lead to a decreased inflammation and a more efficient resolution of this process.

The incorporation of EPA and DHA in humans after supplementation occurs rapidly in plasma fatty acid fractions, between one and four weeks, but in mononuclear cells, it takes months of supplementation [25]. However, in light of the results of the cryo-EM study showing the potential blockage of S-protein by LA, we decided to explore, through in silico simulations, whether other PUFAs could also bind to S-protein (Figure 3) and, therefore, could aid in reducing SARS-CoV-2 infection from two different fronts: by blocking the viral entrance to cells, and via their known effects on inflammation. Analysis of PUFA dietary intake and its relationship with COVID-19 cases, molecular docking, and molecular dynamics studies of S-protein’s predicted complexes and a small library of fatty acids are presented.

## 2. Results and Discussion

### 2.1. Epidemiological-Based Analysis

Several risk factors could explain why some countries have lower incidences and fatality rates than others; some of these factors include population density, economic issues, the strength of public and private health systems, adoption of strict measures such as lockdowns, the presence of comorbidities, and also environmental factors such as diet [26]. In the latter, due to the potential beneficial role of ω-3 PUFA supplementation in Acute Respiratory Distress Syndrome (ARDS), inflammation resolution, coagulation reduction, and arrhythmia improvement [27], it could be considered an important variable in the prognosis of the patients with COVID-19. Part of the rationale for this epidemiological approach was to increase the awareness of actual ω-3 PUFA intake and its possible role on figures related to COVID-19 reported worldwide.

In these analyses, despite the fact that countries with an intake from seafood superior to the 1000 mg/day were reported; such is the case of the Maldives (3886 mg/day), Barbados (1986 mg/day), Seychelles (1291 mg/day), Iceland (1229 mg/day) and Denmark (1225 mg/day), a lower intake of ω-3 PUFA from seafood or marine sources was observed worldwide; 89.2% of the 186 included countries reported an intake <500 mg/day, meanwhile 75.8% and 53.2% were classified with consumption of <250 and <100 mg/day, respectively. Moreover, differences between regions for total cumulative cases per 1 million of the population, fatality rates, and ω-3 intake from marine sources were observed (Table 1). Of these findings the Eastern Mediterranean stands out as the region with a higher mean fatality rate (3.52%) and the lowest ω-3 intake from marine sources (45.14 mg/day), in comparison with South-East Asia with the lowest fatality rate (1.01%) and the highest average consumption (634.00 mg/day).

Furthermore, bivariate analyses were performed, and although a positive correlation between ω-3 intake from plants was observed with total cumulative cases (r_Spearman_ = 0.321; *p* < 0.001), total cumulative cases per 1 million population (r_Spearman_ = 0.329; *p* < 0.001), and fatality rates (r_Spearman_ = 165; *p* > 0.05), a negative trend was observed with ω-3 intake from marine origin, suggesting that the source of ω-3 could be a co-variable associated with these epidemiological measures of frequency (Table 2).

Once the consumption of ω-3 PUFA according to their source was classified as <100, <250, <500 and <1000 mg/day, in agreement to different recommendations [17] and these categories were contrasted with the fatality rate (if they were >2.5 or >4%), no statistical differences were found for the total ingestion of ω-3 (considering ω-3 PUFA from plants and seafood together), nor just ω-3 from plant sources. Nevertheless, among all the nations, 66% and 13.1% of them reported a very low intake of ω-3 PUFA from seafood (<100 mg/day), and also had rates greater than 2.5% (*χ*^2^ = 4.887; *p* = 0.027) and 4% (*χ*^2^ = 9.551; *p* = 0.002), respectively.

**Figure 4:**
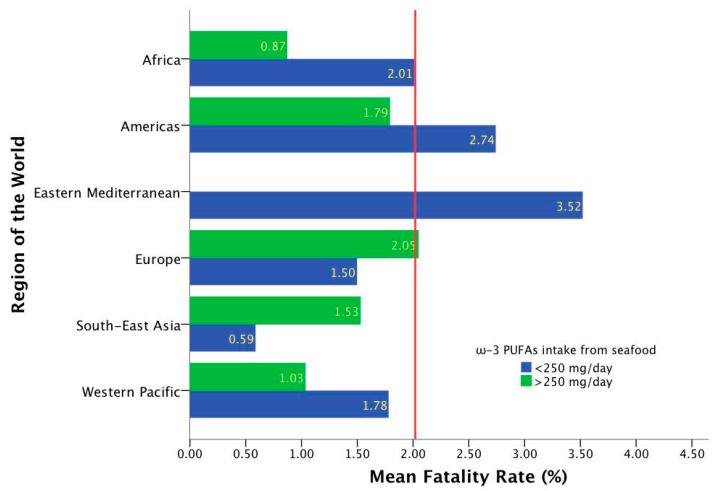
Mean fatality rates according to the region and ω-3 PUFA intake from seafood. The red line represents the global fatality rate (2.02%). Linear regression model considering region and ω-3 PUFA intake: r = 167; r^2^ = 0.028; *p* = 0.074.

Likewise, considering the most common recommendation for ω-3 PUFA, in those nations with a consumption <250 mg/day from marine products, differences among regions were observed (*χ*^2^ = 59.361; *p* = 0.000), as well as a trend for higher fatality rates, >2.5 and 4% (*χ*^2^ = 10.432; *p* = 0.064) and (*χ*^2^ = 10.367; *p* = 0.066), respectively. Additionally, it highlights the case of the Eastern Mediterranean region with the highest mean fatality rate and where any country belonging to this region met this recommendation (Figure 4). On the other hand, the lowest figures in those strata with an adequate intake were reported in Africa, the Americas, and the Western Pacific in comparison with countries of the same group but with low consumption.

This epidemiological approach assessing only one dietetic factor has several limitations, which are important to consider when interpreting the results. The data presented here do not suggested that individuals with ω-3 PUFA deficiency are at an increased risk of COVID-19 infection and mortality, because of the lack of information regarding potential confounders factors such as the accessibility and availability of testing for the disease, contingency measures taken by each country, population characteristics or some other intrinsic factors (ecological fallacy). However, evidence from observational studies, such as this, suggests that the relationship between COVID-19 risk and mortality remains at the level of the individual participants in an environmental context. Furthermore, one strength of this section of the manuscript is the inclusion and analysis of available worldwide data, which provides the opportunity to investigate the differences among countries or regions, and is key information in public health policies, whose object of interest is the groups [28].

### 2.2. In Silico Studies

The results of the epidemiological analysis seem to suggest that there is a certain influence of the nutrient intake, particularly of polyunsaturated fatty acids, in the different fatality rate values that certain regions and countries have had. It was found that linoleic acid (LA), a ω-6 PUFA, could stabilize S-protein’s closed conformation by binding to the fatty acid-binding pocket (FABP); therefore, we decided to explore, through in silico simulations, if other PUFA could attach to this site and potentially have a similar effect as LA. First, we built a small library of 70 fatty acids varying the chain length (from 16 to 24 carbon atoms), the degree of unsaturation (from none to six conjugated double bonds), and the configuration of double bonds (all-*cis* and all-*trans*). Their ability to bind to the FABP was analyzed. This initial exploration suggested that chain length and double bond position were the most important factors: the longer the carbon chain and the presence of ω-3 and ω-6 unsaturation led to higher affinity. Interestingly, the nature of the double bond configuration had a small effect.

Therefore, we decided to study only the *cis*-PUFAs, which are those found in dietary sources. Additionally, we carried out the docking study on the FABP of the open conformation of S-protein. If PUFAs had a better affinity in the open conformation, they could have a weak effect in stabilizing the closed conformation, limiting their potential impact in inhibiting the recognition of the ACE2 receptor. We used two different molecular programs (Molegro Virtual Docker and Yasara Structure, which uses Autodock/Vina algorithms) to find consensus docking poses for further examination. Table 3 shows the results of the theoretical affinity of fatty acids of 20, 22, and 24 carbon atoms to the fatty acid-binding pocket in both closed and open conformations. A complete table can be consulted in the Supplementary Materials. In general, all the studied ligands had a higher affinity to the closed conformation than to the open conformation. From the results in Table 3, long-chain ω-3 PUFAs, including DHA and EPA, have a better potential in stabilizing the closed conformation in comparison to linoleic acid. These results were independent of the docking program used. The root mean square deviation (RMSD) values between the poses predicted by YASARA and Molegro Virtual Docker were lower than 2 Å, giving consensus to carry out further analysis.

**Figure 5:**
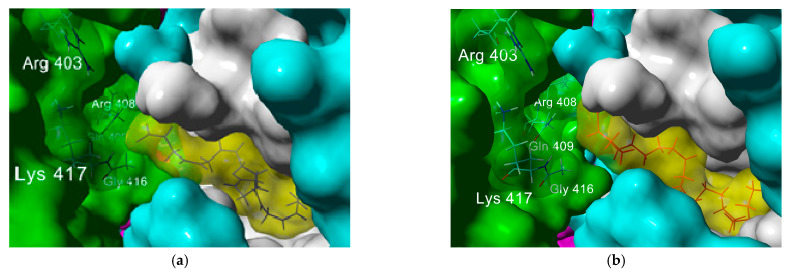
Predicted poses of the complex of (**a**) DHA (unsaturated, in yellow) and (**b**) behenic acid (saturated, in yellow) within the fatty acid-binding pocket of S-protein. Subunit A is colored in blue with the fatty acid binding pocket shown in white. Critical residues for anchoring the carboxylate group to subunit C (colored in green) are labeled.

Some interesting structural features needed for binding in the fatty acid-binding pocket were observed from the predicted poses’ analysis. This site is mostly hydrophobic with five Phe residues that delimit the pocket in a bent tube shape where the carbon tail of ω-3 and ω-6 PUFA fits well; the presence of multiple double bonds allows the hydrocarbon chain to adopt a coiled spring-like conformation, reducing steric clashes within the hindered hydrophobic pocket. On the other hand, the lack of these multiple double bonds in both saturated and ω-9 fatty acids leads to steric hindrance, thus leading to a lower affinity (Figure 5). Additionally, the presence of double bonds could result in the formation of favorable π–π interactions with the aromatic residues within the FABP. The anchor for the carboxylate headgroup is provided by an Arg 408, Gln 409, and Lys 417 from the adjacent trimer, giving rise to a composite fatty acid-binding site [5]. In fact, the interaction of the carboxylate group in PUFA with these basic residues is needed to render a closed conformation: while the long hydrocarbon chain interacts with the highly hydrophobic FABP in one subunit, the ionized carboxylate group interacts through hydrogen bonding and electrostatic interactions with Arg 403, Arg 408, and potentially with Lys 417 in the other subunit (colored in green in Figure 5) closing the RBD and limiting the access to ACE2 receptor. Table 4 shows the distance between the carboxylate group and the aforementioned basic residues; it can be appreciated that the carboxylate group of ω-3 and ω-6 PUFA interacts with residues in both subunits through hydrogen bonding and electrostatic interactions, which results in a more efficient interaction; furthermore, they could be better candidates for RBD allosteric inhibitors.

**Figure 6:**
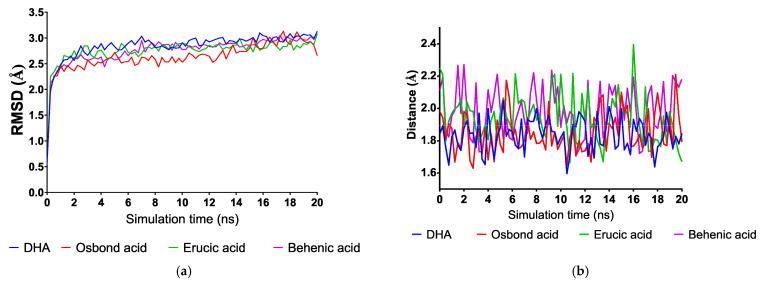
Variations along simulation time of (**a**) root mean square deviation (RMSD) and (**b**) distance from carboxylate groups of the fatty acid to Arg 408 in the adjacent subunit.

The combination of molecular docking and molecular dynamics in silico simulations allows for more realistic approximations during the study of ligand binding against a specific protein. Molecular dynamics incorporate some biological conditions that include structural motions not included in molecular docking procedures; therefore, they help refine the 3D structure of targets and calculate more reliable affinity values [29]. We wanted to analyze, through molecular dynamic simulations, the stability of the complex of DHA (a ω-3 PUFA) and osbond acid (a ω-6 PUFA) in the closed conformation and determine their ligand binding energy via Molecular Mechanics Poisson-Boltzmann Surface Area (MM-PBSA) calculations. We also included erucic acid (ω-9 fatty acid) and behenic acid (a saturated fatty acid) for comparison purposes. The set of plots in Figure 6 show the RMSD variations, ligand binding energies (LBE), and variation of the distance between the oxygen of the fatty acid’s carboxylate group and the amino group of Arg 408’s guanidine group of the four complexes along the simulation time. After a short equilibration time of 0.40 ns, all complexes were stable, with small changes in the RMSD values. More positive values are related to higher affinity in the algorithm implemented in the YASARA structure for LBE calculations. Both DHA and osbond acid had higher LBEs than the ω-9 erucic acid and the saturated behenic acid, thus a higher affinity, which matches the molecular docking results.

As mentioned before, the interaction between the carboxylate group with the basic residues in the subunit adjacent to the fatty acid-binding pocket is needed for RBD blocking. Therefore, we wanted to analyze whether these interactions would be consistent throughout simulation time. As seen in Figure 5b, the distance of one oxygen atom of the carboxylate group to the proton of the guanidino group of Arg 408 is around 2 A along all the simulation time in the four complexes, and slightly shorter for DHA and osbond acid complexes. Hence, we conclude that ω-6, and notably ω-3 PUFA, could be allosteric inhibitors of S-protein and serve as a template for the design of new drugs based on this molecular mechanism.

This pandemic has reminded us of the importance of an effective immune response. It has been proposed that multiple SARS-CoV-2 proteins interact with components of the innate immune system to evade an antiviral response by blocking the activation of type I IFN, the most critical cytokine activated in terms of antiviral protection, leading to uncontrolled virus proliferation [30]; additionally, more neutrophils and macrophages are recruited in the lung tissue, promoting an exacerbated release of pro-inflammatory cytokines, chemokines, and growth factors. These processes lead to a condition reported as “cytokine storm”, which can be responsible for characteristic symptoms of the pulmonary dysfunction in acute lung injury and acute respiratory distress syndrome leading to systemic inflammation and multiple organ failure observed in severe SARS-CoV-2 infected individuals [31]. Although several factors must be considered in the explanation of the variability in the number of cases and fatality rates across regions and countries, based on the results of the epidemiological analysis and the in silico simulations present in this work, we conclude that the intake of ω-3 PUFA from marine origin has had a positive impact for the countries that incorporate them in their diet. In addition to its role in decreasing inflammation and coagulation, ω-3 PUFA could exert some additional therapeutical effect diminishing viral entrance to cells stabilizing the closed conformation of the spike glycoprotein blocking the interaction of ACE2 to the receptor-binding domain. Consequently, there is a need to carry out clinical studies that demonstrate that ω-3 PUFA supplementation could aid and act synergistically with other treatments to promote patient recovery.

## 3. Materials and Methods

### 3.1. Epidemiology Analysis

To address the relationship between ω-3 PUFA dietary intakes (marine vs. plant origin), we retrieved and used reported intake data worldwide [18] and correlated them with the cumulative confirmed cases of COVID-19 and deaths for each country reported by the WHO to 1 December 2020. We computed the case fatality rate as the ratio between deaths and confirmed cases, and restricted the dataset to countries with both information, reporting the data from 186 nations, which were grouped in 6 regions: Africa, Americas, Eastern Mediterranean, Europe, South-East Asia, and Western Pacific, according to the World Health Organization (Table S1) [32]. Additionally, groups according to ω-3 PUFA intake according to origin (marine, plant, and both) were created, considering 100, 250, 500, and 1000 mg/day.

These exploratory analyses were performed with the sole purpose to look for patterns that could suggest hypotheses about the risk or prognostic role of ω-3 PUFA dietary intakes despite the low detailed biological understanding of the COVID-19. Data were analyzed using SPSS software 21.0 (IBM SPSS Inc., Chicago, IL, USA). This included the estimate of descriptive statistics (mean, standard deviation, proportions, and Spearman correlation tests) as well as inferential analyses: one-way analysis of variance with Tukey post-hoc tests among regions and chi-squared (*χ*^2^) to compare strata according to ω-3 PUFA consumption and high fatality rate (>2.5 and >4%). Additionally, a multivariate analysis was made, considering the area and ω-3 PUFA from marine products. Although *p*-values lower than 0.001 were considered as indicative of statistically significant, values less than 0.05 and 0.01 are reported as indicative of trends.

### 3.2. Molecular Docking Studies

The tridimensional structures of different fatty acids were constructed using MarvinSketch (ChemAxon, Inc, Budapest, Hungary), and their structure was further optimized using MMFF94 force field. The docking studies were carried out using the crystal structure of the SARS-CoV-2 S-protein in its closed conformation and bound to LA (PDB code: 6ZB5 [11]) and in its open conformation (PDB code: 7CAI [10]). Protein structures were retrieved from the Protein Data Bank [33]. All co-crystallized ligands were removed from the downloaded structures. Docking studies were carried out in Molegro Virtual Docker v.6.0.1 [34] (Qiagen Bioinformatics, Aarhus, Denmark) and Yasara Structure v.18.4.24 [35,36] (Yasara, Gmb, Austria) using the standard protocol referred by the fabricant with 35 runs. Yasara Structure uses the searching algorithms of AutoDock VINA [37] with its scoring scheme based on ligand binding energy. The fatty acid-binding pocket was selected as the searching site and delimited by a 15 Å sphere in Molegro Virtual Docker or a 15 Å cube in Yasara Structure. The fatty acid-binding pocket is mostly hydrophobic, defined by five Phe residues (Phe338, Phe342, Phe374, Phe377, and Phe392) that form a bent tube. In the closed conformation, the anchor for the headgroup carboxyl is provided by two polar residues, Arg408 and Gln409 from the adjacent RBD in the trimer, giving rise to a composite fatty acid-binding site [11]. The assignments of charges on each protein and the analyzed ligands were based on each program’s standard templates. The structures with the highest theoretical affinity of each compound were selected for further analysis. To evaluate this procedure’s efficacy, the co-crystallized linoleic acid in SARS-CoV-2 protein spike S was also included in the docking study of its respective receptor; the RMSD of the position of the highest theoretical affinity was lower than 2 Å (1.87 Å). The analysis for consensus positions was carried out in Yasara Structure, aligning the predicted structures by Molegro Virtual Docker and Yasara using the MUSTANG tool.

### 3.3. Molecular Dynamics Simulations

Molecular Dynamics (MD) simulations were carried out to observe the stability of the predicted complexes of the fatty acids 22 carbon chains with the S-protein in its closed conformation. Simulations were performed in Yasara Structure v.18.4.24 [36] using AMBER14 force field [38]. The initial structures for the MD simulations were obtained from the docking complexes of DHA, osbond acid, erucic acid, and behenic acid generated with Yasara Structure, because they had minor variations with those predicted by Molegro Virtual Docker. Each complex was located within a water box with a size of 100 Å × 100 Å × 100 Å, with periodic boundary conditions. The temperature was set at 298 K, water density to 0.997 g/cm^3^, and pH to 7.4; sodium (Na^+^) and chlorine (Cl^−^) ions were included for charge neutralization and to simulate physiological conditions. A particle mesh Ewald (PME) algorithm was applied with a cut-off radius of 8 Å. A time step of 2.5 fs was set. A total simulation time of 20 ns was enough to achieve convergence, because RMSD variations were lower than 0.5 Å for 19 ns. Snapshots were recorded at intervals of 200 ps. Results were analyzed with a script included as part of YASARA software and included the root mean square deviation (RMSD) and ligand binding energy (LBE) using MM-PBSA calculations.

## Figures and Tables

**Table 1:** Epidemiological data of COVID-19 and estimated ω-3 PUFA intake: comparison among regions worldwide.

Region According to WHO (Number of Included Countries)	Total Cumulative Cases ^1^ *n* = 186	Total Cumulative Cases per 1 Million Population ^1^ *n* = 186	Fatality Rates ^1^ *n* = 186	ω-3 Marine Source Intake (mg/day) *n* = 186	ω-3 Plant Source Intake (mg/day) *n* = 186	ω-3 Marine + Plants Intake (mg/day) *n* = 186
Global (186)	336,937.59 ± 116,734.903	11,334.15 ± 14,635.058	2.02 ± 2.494	216.05 ± 380.148	838.81 ± 636.988	1054.86 ± 692.244
Africa (46)	32,392.63 ± 116,734.903	1895.015 ± 3441.064	1.94 ± 1.297	118.41 ± 195.488	726.96 ± 523.41	847.369 ± 576.333
Americas (34)	781,579.21 ± 2,466,433.13	12,101.95 ± 11,971.255	2.60 ± 1.889	164.26 ± 334.524	1005.29 ± 938.574	1169.55 ± 942.427
Eastern Mediterranean (22)	186,972.68 ± 225,982.552	14,092.86 ± 14,666.749	3.52 ± 6.036	45.14 ± 23.77	1027.45 ± 504.376	1072.59 ± 510.274
Europe (51)	268,574.41 ± 564,798.905	24,162.05 ± 16,505.756	1.65 ± 0.835	232.27 ± 254.529	904.27 ± 477.182	1136.55 ± 556.833
South-East Asia (11)	984,590.45 ± 2,818,704.55	4280.50 ± 7106.939	1.01 ± 1.017	634.00 ± 1132.984	517.27 ± 485.418	1151.27 ± 1094.051
Western Pacific (22)	39,338.55 ± 95,821.332	914.59 ± 2221.254	1.17 ± 1.565	424.59 ± 236.084	631.59 ± 690.112	1056.18 ± 639.115
*p* ^2^	0.058	0.000 ***^,a,b,f,h,i,j,k,l,m^	0.008 *^,a,b^	0.000 ***^,b,c,d,e,f,g^	0.044 *	0.307

Data are presented as mean ± standard deviation; ^1^ Official figures reported by WHO as of 1 December 2020; ^2^ Comparison test ANOVA * *p* < 0.05; *** *p* < 0.001. Tukey’s post-hoc comparison; ^a^ Eastern Mediterranean vs. Europe; ^b^ Eastern Mediterranean vs. Western Pacific; ^c^ Africa vs. South-East Asia; ^d^ Americas vs. South-East Asia; ^e^ Eastern Mediterranean vs. South-East Asia; ^f^ South-East Asia vs. Europe; ^g^ Western Pacific vs. Africa; ^h^ Africa vs. Americas; ^i^ Africa vs. Eastern Mediterranean; ^j^ Africa vs. Europe; ^k^ Americas vs. Europe; ^l^ Americas vs. Western Pacific; ^m^ Europe vs. Western Pacific.

**Table 2:** Correlation matrix ω-3 PUFA intake and epidemiological figures by COVID-19 ^1^.

ω-3 Source Intake	Total Cumulative Cases	Total Cumulative Cases per 1 Million Population	Fatality Rates
ω-3 marine and plants	0.219 **	0.257 **	0.041
ω-3 marine source	−0.087	0.020	−0.141
ω-3 plants source	0.321 ***	0.329 ***	0.165 *

^1^ Official figures reported by WHO as 1 December 2020; Data are presented as Spearman’s rho; * *p* < 0.05; ** *p* < 0.01; *** *p* < 0.001.

**Table 3:** Selected results from the docking study carried out on S-protein in its closed and open conformations ^1^.

		Yasara Structure (Kcal/mol)		Molegro Virtual Docker	
Common Name	Fatty Acid Type	Closed	Open	Closed	Open
Tetracosahexaenoic acid (Nisinic acid)	24:6 (ω-3)	9.43	4.78	−126.7	−91.2
Tetracosapentaenoic acid	24:5 (ω-6)	8.58	4.53	−124.3	−97.0
Nervonic acid	24:1 (ω-9)	7.91	4.41	−115.9	−73.7
Docosahexaenoic acid (DHA)	22:6 (ω-3)	8.87	4.72	−120.1	−85.3
Docosapentaenoic acid (Osbond acid)	22:5 (ω-6)	8.69	4.84	−117.9	−77.9
Erucic acid	22:1 (ω-9)	7.72	4.49	−104.9	−71.5
Eicosapentaenoic acid (EPA)	20:5 (ω-3)	8.58	5.55	−110.8	−88.2
Arachidonic acid (AA)	20:4 (ω-6)	8.23	4.80	−116.1	−82.3
Gondoic acid	20:1 (ω-9)	7.44	5.11	−106.3	−79.9
Lignoceric acid	24:0	8.01	4.10	−110.2	−72.3
Behenic acid	22:0	7.37	4.09	−109.1	−72.1
Arachidic acid	20:0	6.59	4.19	−108.2	−73.1
Linoleic acid	18:2 (ω-6)	6.79	4.37	−104.8	−79.6

^1^ Higher theoretical affinity. Is indicated by more positive score values in Yasara Structure and more negative score values in Molegro Virtual Docker.

**Table 4:** Distance (Å) of the hydrogen bonds and electrostatic interactions from the carboxylate group in fatty acids with 22 carbon chains.

	Hydrogen Bond				Electrostatic	
	Phe 374A	Arg 403C	Asp 405C	Gln 409C	Arg 403C	Arg 408C
Erucic acid (ω-9)	n.a.	n.a.	n.a.	n.a.	n.a.	n.a.
Osbond acid (ω-6)	3.21	2.94	2.06	2.88	4.85	n.a.
Docoesahexaenoic acid (ω-3)	3.53	3.11	1.94	2.09	4.85	n.a.
Behenic acid	1.89	n.a.	n.a.	2.03	n.a.	3.7

n.a. Absence of polar interaction with these residues.

## Data Availability

Data is available in the supplementary materials.

## Supplementary Materials

The following are available online, Table S1: Data from epidemiological studies; Table S2: Table with full docking results.
