# Supplementary material for: In Silico Study of Polyunsaturated Fatty Acids as Potential SARS-CoV-2 Spike Protein Closed Conformation Stabilizers: Epidemiological and Computational Approaches
Source: Molecules. 2021 Jan 29;26(3):711. doi: 10.3390/molecules26030711 (PMC7866518; doi:10.3390/molecules26030711)
Supplement: Supplementary file 1 [file molecules-26-00711-s001.zip › Table S1 (full epidemiological).pdf]

Table S1. Epidemiological data COVID-19 and omega 3 intake by source, country, region and globally *

Excluded for analyses. Incompleted data	Name	WHO Region	Seafood omega-3 mg/day (Uncertain Intervals)	Plant omega-3 mg/day (Uncertain Intervals)	Total omega-3 (seafood + plant) mg/day	Cases - cumulative total	Cases - cumulative total per 1 million population	Deaths - cumulative total	Fatality rate
	Global		163 (154, 172)	1371 (1299, 1465)	1534.0	62844837	8050.517041	1465144	2.331367333
	Afghanistan	Eastern Mediterranea	30 (18, 49)	665 (325, 1181)	695.0	46498	1194.45	1774	3.815217859
	Albania	Europe	47 (26, 75)	563 (266, 1075)	610.0	38182	13267.79	810	2.121418469
	Algeria	Africa	42 (27, 64)	2180 (1059, 4027)	2222.0	83199	1897.31	2431	2.921910119
Excluded	American Samoa	Western Pacific						0	0
	Andorra	Europe	324 (192, 509)	633 (311, 1165)	957.0	6745	87296.96	76	1.126760563
	Angola	Africa	87 (49, 141)	2195 (1048, 3993)	2282.0	15139	460.62	348	2.298698725
Excluded	Anguilla	Americas				6	399.95	0	0
	Antigua and Barbuda	Americas	310 (166, 510)	1059 (538, 1955)	1369.0	141	1439.82	4	2.836879433
	Argentina	Americas	48 (27, 77)	1304 (653, 2344)	1352.0	1418807	31392.47	38473	2.71164436
	Armenia	Europe	41 (24, 68)	466 (231, 850)	507.0	135967	45884.53	2193	1.612891363
Excluded	Aruba	Americas				4838	45314.05	45	0.93013642
	Australia	Western Pacific	286 (271, 302)	914 (458, 1658)	1200.0	27904	1094.28	908	3.254013761
	Austria	Europe	184 (110, 294)	738 (359, 1372)	922.0	280530	31147.86	2983	1.063344384
	Azerbaijan	Europe	37 (21, 59)	342 (161, 660)	379.0	121176	11951.27	1392	1.148742325
	Bahamas	Americas	169 (100, 275)	405 (192, 763)	574.0	7517	19115.36	163	2.168418252
	Bahrain	Eastern Mediterranea	52 (29, 86)	1451 (684, 2694)	1503.0	86956	51103.24	341	0.392152353
	Bangladesh	South-East Asia	47 (34, 61)	431 (203, 759)	478.0	464932	2823.08	6644	1.42902618
	Barbados	Americas	1986 (1623, 2391)	165 (152, 177)	2151.0	276	960.42	7	2.536231884
	Belarus	Europe	116 (67, 191)	944 (455, 1763)	1060.0	136647	14461.04	1158	0.847439022
	Belgium	Europe	281 (247, 323)	590 (298, 1063)	871.0	577249	49807.4	16645	2.883504346
	Belize	Americas	73 (43, 113)	490 (249, 881)	563.0	5743	14443.15	147	2.55963782
	Benin	Africa	75 (44, 122)	520 (260, 946)	595.0	3015	248.7	43	1.426202322
Excluded	Bermuda	Americas				260	4175.16	9	3.461538462
	Bhutan	South-East Asia	23 (14, 37)	460 (231, 846)	483.0	410	531.36	0	0
	Bolivia (Plurinational State of	Americas	16 (8, 26)	545 (267, 1007)	561.0	144622	12389.42	8952	6.18992961
Excluded	Bonaire, Sint Eustatius and Sa	Americas				162	6178.25	3	1.851851852
	Bosnia and Herzegovina	Europe	47 (26, 75)	563 (266, 1075)	610.0	87901	26792.4	2681	3.050022184
	Botswana	Africa	10 (6, 16)	387 (187, 708)	397.0	9992	4248.97	31	0.310248199
	Brazil	Americas	57 (54, 60)	1747 (1658, 1846)	1804.0	6314740	29708.12	172833	2.736977294
Excluded	British Virgin Islands	Americas				73	2414.26	1	1.369863014
	Brunei Darussalam	Western Pacific	239 (140, 381)	290 (138, 548)	529.0	150	342.87	3	2
	Bulgaria	Europe	119 (93, 149)	1444 (719, 2752)	1563.0	145300	20911.15	4035	2.777013076
	Burkina Faso	Africa	22 (15, 31)	498 (239, 925)	520.0	2886	138.06	68	2.356202356
	Burundi	Africa	25 (14, 41)	164 (73, 306)	189.0	689	57.94	1	0.145137881
	Cabo Verde	Africa	120 (69, 189)	1232 (594, 2288)	1352.0	10526	18932.1	104	0.988029641
	Cambodia	Western Pacific	680 (395, 1148)	215 (105, 386)	895.0	326	19.5	0	0
	Cameroon	Africa	142 (80, 240)	503 (246, 915)	645.0	24487	922.44	441	1.800955609
	Canada	Americas	92 (82, 103)	2085 (1925, 2249)	2177.0	370278	9810.73	12032	3.249450413
Excluded	Cayman Islands	Americas				274	4169.2	2	0.729927007
	Central African Republic	Africa	38 (21, 62)	1529 (717, 2863)	1567.0	4918	1018.27	63	1.28100854
	Chad	Africa	72 (41, 113)	771 (358, 1468)	843.0	1688	102.76	101	5.983412322
	Chile	Americas	407 (226, 695)	1230 (615, 2141)	1637.0	551743	28862.59	15410	2.792967015
	China	Western Pacific	37 (33, 41)	3266 (3089, 3448)	3303.0	93577	63.6	4750	5.076033641
	Colombia	Americas	94 (86, 101)	510 (251, 889)	604.0	1308376	25713.48	36584	2.796138113
	Comoros	Africa	243 (133, 408)	126 (58, 237)	369.0	611	702.62	7	1.145662848
	Congo	Africa	148 (82, 249)	1067 (507, 1975)	1215.0	5774	1046.38	94	1.62798753
Excluded	Cook Islands	Western Pacific				0	0	0	0
	Costa Rica	Americas	54 (31, 87)	621 (311, 1156)	675.0	137093	26912.02	1690	1.232739819
	Côte d'Ivoire	Africa	140 (83, 223)	205 (98, 382)	345.0	21331	808.66	132	0.618817683
	Croatia	Europe	145 (100, 203)	1196 (578, 2262)	1341.0	128442	31287.12	1786	1.390510892
	Cuba	Americas	62 (36, 102)	836 (411, 1579)	898.0	8284	731.37	135	1.629647513
Excluded	CuraÃ§ao	Americas				2364	14406.46	4	0.169204738
	Cyprus	Europe	213 (149, 298)	741 (367, 1319)	954.0	10565	8750.5	49	0.463795551
	Czechia	Europe	113 (65, 176)	1364 (650, 2562)	1477.0	523298	48865.34	8295	1.585138869
	Democratic People's Republic	South-East Asia	23 (13, 37)	1899 (919, 3706)	1922.0	0	0	0	0
	Democratic Republic of the C	Africa	62 (36, 98)	382 (180, 687)	444.0	12771	142.59	333	2.607470049
	Denmark	Europe	1225 (679, 2013)	300 (142, 558)	1525.0	80481	13894.72	837	1.039997018
	Djibouti	Eastern Mediterranea	13 (7, 22)	640 (332, 1175)	653.0	5679	5747.98	61	1.07413277
	Dominica	Americas	154 (84, 260)	485 (242, 905)	639.0	85	1180.7	0	0
	Dominican Republic	Americas	54 (33, 86)	1566 (774, 2952)	1620.0	143988	13273.34	2331	1.618884907
	Ecuador	Americas	36 (21, 58)	1027 (516, 1903)	1063.0	192685	10921.3	13461	6.986013442
	Egypt	Eastern Mediterranea	77 (49, 112)	568 (273, 1076)	645.0	115911	1132.67	6650	5.737160408
	El Salvador	Americas	45 (26, 74)	453 (220, 795)	498.0	38405	5921.03	1114	2.900663976
	Equatorial Guinea	Africa	94 (55, 157)	1391 (678, 2545)	1485.0	5153	3672.88	85	1.649524549
	Eritrea	Africa	11 (6, 18)	406 (205, 749)	417.0	577	162.7	0	0
	Estonia	Europe	188 (162, 217)	1217 (615, 2261)	1405.0	12308	9278.31	118	0.958726032
	Eswatini	Africa	18 (10, 29)	280 (135, 540)	298.0	6419	5532.84	122	1.900607571
	Ethiopia	Africa	48 (28, 80)	240 (111, 437)	288.0	110074	957.47	1706	1.549866453
Excluded	Falkland Islands (Malvinas)	Americas				17	4880.85	0	0
Excluded	Faroe Islands	Europe				503	10293.67	0	0
	Fiji	Western Pacific	321 (180, 521)	1258 (603, 2382)	1579.0	42	46.85	2	4.761904762
	Finland	Europe	511 (436, 603)	2014 (1842, 2200)	2525.0	24912	4496.17	399	1.601637765
	France	Europe	404 (239, 636)	736 (364, 1404)	1140.0	2183275	33448.1	52371	2.398735844
Excluded	French Guiana	Americas				11204	37511.47	70	0.624776865
Excluded	French Polynesia	Western Pacific				14372	51162.66	75	0.521848038
	Gabon	Africa	199 (109, 343)	925 (466, 1783)	1124.0	9191	4129.42	59	0.641932325
	Gambia	Africa	196 (115, 319)	1336 (643, 2529)	1532.0	3742	1548.41	123	3.287012293
	Georgia	Europe	112 (64, 189)	379 (195, 680)	491.0	139343	34930.35	1303	0.935102589
	Germany	Europe	226 (189, 267)	1404 (1319, 1500)	1630.0	1067473	12740.78	16636	1.558446912
	Ghana	Africa	268 (149, 446)	569 (276, 1090)	837.0	51667	1662.77	323	0.625157257
Excluded	Gibraltar	Europe				1014	30097.06	5	0.493096647
	Greece	Europe	209 (181, 239)	400 (195, 750)	609.0	105271	10099.82	2406	2.285529728
Excluded	Greenland	Europe				18	317.06	0	0
	Grenada	Americas	254 (136, 407)	1069 (518, 1947)	1323.0	41	364.37	0	0
Excluded	Guadeloupe	Americas				8378	20938.51	149	1.778467415
Excluded	Guam	Western Pacific				6722	39828.17	112	1.666170783
	Guatemala	Americas	21 (12, 36)	703 (335, 1263)	724.0	122062	6813.18	4171	3.417115892
Excluded	Guernsey	Europe				286	4525.6	13	4.545454545
	Guinea	Africa	92 (52, 149)	968 (472, 1831)	1060.0	13097	997.27	76	0.580285562
	Guinea-Bissau	Africa	10 (5, 17)	1079 (518, 1951)	1089.0	2422	1230.69	43	1.775392238
	Guyana	Americas	228 (127, 381)	249 (122, 461)	477.0	5376	6834.89	150	2.790178571
	Haiti	Americas	36 (20, 57)	841 (412, 1615)	877.0	9294	815.08	233	2.506993759
Excluded	Holy See	Europe				26	32138.44	0	0
	Honduras	Americas	29 (17, 48)	153 (74, 273)	182.0	107888	10892.71	2909	2.696314697
	Hungary	Europe	72 (55, 93)	562 (526, 604)	634.0	221073	22884.57	4977	2.251292559
	Iceland	Europe	1229 (947, 1557)	1271 (1141, 1415)	2500.0	5392	15801.06	26	0.482195846
	India	South-East Asia	31 (18, 48)	552 (270, 1002)	583.0	9462809	6857.09	137621	1.4543356
	Indonesia	South-East Asia	755 (689, 829)	256 (243, 269)	1011.0	538883	1970.15	16945	3.144467352
	Iran (Islamic Republic of)	Eastern Mediterranea	50 (38, 64)	1195 (559, 2257)	1245.0	962070	11454.18	48246	5.014811812
	Iraq	Eastern Mediterranea	44 (25, 70)	922 (462, 1674)	966.0	552549	13737.31	12258	2.218445785
	Ireland	Europe	97 (66, 138)	626 (295, 1115)	723.0	72544	14691.6	2053	2.830006617
Excluded	Isle of Man	Europe				369	4339.54	25	6.775067751
	Israel	Europe	232 (198, 269)	2 (2, 2)	234.0	335919	38809.73	2864	0.852586487
	Italy	Europe	281 (213, 358)	713 (346, 1257)	994.0	1601554	26488.68	55576	3.470129637
	Jamaica	Americas	82 (68, 99)	5542 (5176, 5945)	5624.0	10763	3634.72	257	2.38781009
	Japan	Western Pacific	718 (629, 817)	1224 (1164, 1287)	1942.0	148694	1175.67	2139	1.438524756
Excluded	Jersey	Europe				923	8483.46	32	3.46695558
	Jordan	Eastern Mediterranea	47 (28, 77)	1410 (665, 2645)	1457.0	219430	21506.14	2751	1.253702775
	Kazakhstan	Europe	59 (33, 96)	870 (416, 1580)	929.0	173806	9256.47	2477	1.425152181
	Kenya	Africa	41 (23, 65)	217 (101, 399)	258.0	83618	1555.07	1469	1.756798775
	Kiribati	Western Pacific	496 (292, 804)	183 (88, 345)	679.0	0	0	0	0
Excluded	Kosovo[1]	Europe				39553	21260.7	1006	2.543422749
	Kuwait	Eastern Mediterranea	76 (43, 127)	1159 (575, 2228)	1235.0	142635	33399.51	880	0.616959372
	Kyrgyzstan	Europe	44 (26, 72)	397 (191, 734)	441.0	73178	11216.4	1275	1.742326929
	Lao People's Democratic Rep	Western Pacific	489 (275, 776)	223 (107, 416)	712.0	39	5.36	0	0
	Latvia	Europe	180 (158, 207)	1665 (774, 3203)	1845.0	17075	9052.6	206	1.206442167
	Lebanon	Eastern Mediterranea	8 (7, 10)	1918 (946, 3595)	1926.0	127903	18739.14	1018	0.795915655
	Lesotho	Africa	10 (6, 17)	169 (78, 327)	179.0	2109	984.48	44	2.086296823

	Liberia	Africa	51 (29, 85)	461 (224, 867)	512.0	1598	315.96	83	5.193992491
	Libya	Eastern Mediterranean	60 (35, 101)	1265 (632, 2343)	1325.0	82809	12051.45	1183	1.42858868
Excluded	Liechtenstein	Europe				1302	34140.07	15	1.152073733
	Lithuania	Europe	230 (194, 270)	1253 (628, 2276)	1483.0	62515	22964.13	519	0.830200752
	Luxembourg	Europe	233 (159, 328)	390 (186, 724)	623.0	34538	55174.46	312	0.903352829
	Madagascar	Africa	72 (44, 120)	590 (285, 1093)	662.0	17341	626.23	251	1.447436711
	Malawi	Africa	52 (29, 85)	393 (195, 746)	445.0	6028	315.11	185	3.069011281
	Malaysia	Western Pacific	988 (559, 1613)	387 (189, 737)	1375.0	65697	2029.82	360	0.547970227
	Maldives	South-East Asia	3886 (2106, 6641)	215 (106, 392)	4101.0	13011	24070.2	46	0.353546999
	Mali	Africa	76 (53, 103)	667 (327, 1228)	743.0	4688	231.5	152	3.242320819
	Malta	Europe	279 (217, 362)	402 (200, 780)	681.0	9873	22360.22	137	1.38762281
	Marshall Islands	Western Pacific	388 (225, 629)	430 (207, 820)	818.0	4	67.57	0	0
Excluded	Martinique	Americas				5486	14619	40	0.729128691
	Mauritania	Africa	142 (78, 235)	832 (404, 1532)	974.0	8424	1811.75	172	2.041785375
	Mauritius	Africa	395 (224, 609)	1326 (634, 2558)	1721.0	504	396.3	10	1.984126984
Excluded	Mayotte	Africa				5181	18990.89	49	0.945763366
	Mexico	Americas	39 (36, 43)	464 (444, 485)	503.0	1107071	8586.42	105655	9.543651672
	Micronesia (Federated States	Western Pacific	368 (216, 575)	374 (179, 696)	742.0	0	0	0	0
Excluded	Monaco	Europe				609	15518.3	3	0.492610837
	Mongolia	Western Pacific	15 (9, 25)	557 (282, 1011)	572.0	791	241.28	0	0
	Montenegro	Europe	75 (50, 106)	293 (139, 566)	368.0	35366	56309.37	499	1.410959679
Excluded	Montserrat	Americas				13	2600.52	1	7.692307692
	Morocco	Eastern Mediterranean	63 (36, 98)	1283 (614, 2274)	1346.0	356335	9654.01	5846	1.640591017
	Mozambique	Africa	25 (23, 28)	679 (330, 1249)	704.0	15701	502.34	131	0.834341762
	Myanmar	South-East Asia	620 (360, 1034)	593 (282, 1127)	1213.0	89486	1644.67	1918	2.143352033
	Namibia	Africa	23 (13, 37)	233 (115, 450)	256.0	14380	5659.4	152	1.057023644
Excluded	Nauru	Western Pacific				0	0	0	0
	Nepal	South-East Asia	20 (15, 27)	468 (233, 936)	488.0	233452	8012.27	1508	0.645957199
	Netherlands	Europe	180 (158, 202)	640 (316, 1179)	820.0	522835	30512.92	9363	1.790813545
Excluded	New Caledonia	Western Pacific				33	115.59	0	0
	New Zealand	Western Pacific	374 (215, 602)	1041 (497, 1970)	1415.0	1703	353.16	25	1.467997651
	Nicaragua	Americas	37 (21, 62)	542 (270, 998)	579.0	4629	698.76	160	3.45647008
	Niger	Africa	28 (15, 47)	389 (187, 721)	417.0	1548	63.95	72	4.651162791
	Nigeria	Africa	64 (37, 106)	834 (416, 1514)	898.0	67557	327.72	1173	1.736311559
Excluded	Niue	Western Pacific				0	0	0	0
	North Macedonia	Europe	59 (34, 95)	1316 (627, 2495)	1375.0	61878	29700.77	1763	2.849154788
Excluded	Northern Mariana Islands (Co	Western Pacific				106	1841.65	2	1.886792453
	Norway	Europe	596 (518, 683)	1076 (1004, 1152)	1672.0	35826	6608.45	332	0.926701278
	occupied Palestinian territory	Eastern Mediterranean	8 (4, 14)	973 (474, 1789)	981.0	98850	19376.98	822	0.831562974
	Oman	Eastern Mediterranean	45 (25, 74)	1181 (553, 2282)	1226.0	123699	24223.23	1423	1.150373083
Excluded	Other	Other				741		13	1.754385965
	Pakistan	Eastern Mediterranean	16 (9, 26)	277 (136, 514)	293.0	398024	1801.89	8025	2.016210078
Excluded	Palau	Western Pacific				0	0	0	0
	Panama	Americas	130 (75, 210)	667 (329, 1215)	797.0	164729	38177.96	3060	1.857596416
	Papua New Guinea	Western Pacific	294 (164, 493)	303 (146, 586)	597.0	656	73.32	7	1.067073171
	Paraguay	Americas	62 (36, 101)	1575 (800, 2964)	1637.0	81906	11483.43	1743	2.128049227
	Peru	Americas	147 (85, 241)	807 (407, 1506)	954.0	962530	29192.47	35923	3.732143414
	Philippines	Western Pacific	590 (533, 650)	131 (62, 244)	721.0	431630	3938.91	8392	1.944257813
Excluded	Pitcairn Islands	Western Pacific				0	0	0	0
	Poland	Europe	107 (86, 133)	1250 (1156, 1362)	1357.0	990811	26179.65	17150	1.730905289
	Portugal	Europe	582 (472, 711)	604 (297, 1095)	1186.0	298061	29231.1	4505	1.511435579
Excluded	Puerto Rico	Americas				52545	18366.9	1106	2.104862499
	Qatar	Eastern Mediterranean	50 (27, 83)	1505 (682, 2900)	1555.0	138833	48188.28	237	0.170708693
Excluded	RÃ©union	Africa				8054	8995.75	40	0.496647629
	Republic of Korea	Western Pacific	708 (633, 796)	883 (421, 1609)	1591.0	34652	675.88	526	1.517949902
	Republic of Moldova	Europe	108 (61, 173)	613 (297, 1108)	721.0	107364	26615.02	2304	2.145970716
	Romania	Europe	61 (34, 97)	1335 (642, 2401)	1396.0	475362	24709.93	11331	2.383657087
	Russian Federation	Europe	180 (155, 206)	992 (487, 1867)	1172.0	2322056	15911.64	40464	1.742593633
	Rwanda	Africa	18 (10, 29)	303 (157, 539)	321.0	5934	458.15	49	0.825749916
Excluded	Saint BarthÃ©lemy	Americas				152	15376.83	0	0
Excluded	Saint Helena	Africa				0	0	0	0
Excluded	Saint Kitts and Nevis	Americas				22	413.6	0	0
	Saint Lucia	Americas	263 (156, 427)	129 (55, 254)	392.0	257	1399.58	2	0.778210117
Excluded	Saint Martin	Americas				744	19245.2	12	1.612903226
Excluded	Saint Pierre and Miquelon	Americas				14	2415.88	0	0
	Saint Vincent and the Grenad	Americas	114 (66, 184)	702 (335, 1304)	816.0	85	766.18	0	0
	Samoa	Western Pacific	521 (312, 837)	152 (77, 288)	673.0	0	0	0	0
Excluded	San Marino	Europe				1612	47498.38	46	2.853598015
	Sao Tome and Principe	Africa	200 (115, 318)	792 (385, 1531)	992.0	991	4521.83	17	1.715438951
	Saudi Arabia	Eastern Mediterranean	58 (34, 93)	656 (328, 1193)	714.0	357360	10264.87	5896	1.649876875
	Senegal	Africa	229 (126, 372)	2183 (1046, 3958)	2412.0	16089	960.89	333	2.069737087
	Serbia	Europe	57 (37, 82)	1020 (515, 1825)	1077.0	175438	25192.98	1604	0.914283109
	Seychelles	Africa	1291 (740, 2144)	637 (301, 1203)	1928.0	172	1748.91	0	0
	Sierra Leone	Africa	205 (115, 346)	487 (239, 884)	692.0	2412	302.37	74	3.067993367
	Singapore	Western Pacific	49 (44, 54)	887 (454, 1621)	936.0	58218	9951.21	29	0.049812773
Excluded	Sint Maarten	Americas				1066	24858.92	25	2.34521576
	Slovakia	Europe	59 (39, 86)	1253 (609, 2252)	1312.0	105929	19402.19	839	0.792039951
	Slovenia	Europe	69 (45, 101)	1355 (665, 2535)	1424.0	75814	36467.66	948	1.250428681
	Solomon Islands	Western Pacific	412 (238, 668)	102 (47, 191)	514.0	17	24.75	0	0
	Somalia	Eastern Mediterranean	48 (27, 79)	337 (160, 614)	385.0	4451	280.06	113	2.538755336
	South Africa	Africa	14 (13, 15)	683 (652, 717)	697.0	790004	13320.21	21535	2.725935565
Excluded	South Sudan	Africa				3109	277.74	61	1.962045674
	Spain	Europe	647 (512, 807)	874 (414, 1556)	1521.0	1648187	35251.73	45069	2.734459136
	Sri Lanka	South-East Asia	720 (401, 1158)	106 (52, 189)	826.0	23484	1096.7	116	0.49395333
	Sudan	Eastern Mediterranean	17 (9, 26)	526 (254, 981)	543.0	17810	406.16	1249	7.012914093
	Suriname	Americas	133 (75, 222)	1308 (623, 2389)	1441.0	5312	9055.08	117	2.202560241
	Sweden	Europe	400 (348, 466)	1355 (1267, 1459)	1755.0	243129	24073.93	6681	2.747923942
	Switzerland	Europe	212 (120, 339)	508 (241, 947)	720.0	325770	37641.16	4430	1.359855113
	Syrian Arab Republic	Eastern Mediterranean	17 (10, 28)	1134 (552, 2061)	1151.0	7887	450.67	417	5.287181438
	Tajikistan	Europe	19 (11, 32)	1134 (562, 2138)	1153.0	12194	1278.51	86	0.705264884
	Thailand	South-East Asia	824 (477, 1349)	506 (242, 961)	1330.0	3998	57.28	60	1.500750375
	The United Kingdom	Europe	318 (268, 371)	2414 (2261, 2568)	2732.0	1629661	24005.84	58448	3.586512778
	Timor-Leste	South-East Asia	25 (12, 47)	204 (100, 369)	229.0	30	22.75	0	0
	Togo	Africa	94 (53, 153)	566 (262, 1050)	660.0	2946	355.85	64	2.172437203
Excluded	Tokelau	Western Pacific				0	0	0	0
	Tonga	Western Pacific	441 (257, 725)	464 (226, 874)	905.0	0	0	0	0
	Trinidad and Tobago	Americas	22 (19, 25)	996 (482, 1857)	1018.0	6660	4758.88	120	1.801801802
	Tunisia	Eastern Mediterranean	84 (49, 135)	2215 (1096, 4056)	2299.0	96769	8187.84	3260	3.368847461
	Turkey	Europe	377 (304, 459)	1501 (1381, 1648)	1878.0	500865	5938.71	13746	2.744452098
	Turkmenistan	Europe	67 (37, 113)	570 (275, 1058)	637.0	0	0	0	0
Excluded	Turks and Caicos Islands	Americas				748	19319.18	6	0.802139037
Excluded	Tuvalu	Western Pacific				0	0	0	0
	Uganda	Africa	113 (63, 185)	557 (267, 1028)	670.0	20459	447.28	205	1.002004008
	Ukraine	Europe	180 (103, 287)	1057 (524, 1916)	1237.0	745123	17037.71	12548	1.68401727
	United Arab Emirates	Eastern Mediterranean	78 (43, 137)	962 (446, 1835)	1040.0	168860	17073.12	572	0.338742153
	United Republic of Tanzania	Africa	69 (40, 111)	339 (167, 641)	408.0	509	8.52	21	4.125736739
	United States of America	Americas	141 (128, 157)	1527 (1456, 1599)	1668.0	13234551	39983.22	264808	2.000883899
Excluded	United States Virgin Islands	Americas				1544	14785.73	23	1.489637306
	Uruguay	Americas	69 (39, 113)	1384 (661, 2546)	1453.0	5716	1645.49	76	1.32960112
	Uzbekistan	Europe	15 (9, 24)	773 (374, 1428)	788.0	73094	2183.92	610	0.834541823
	Vanuatu	Western Pacific	353 (202, 577)	333 (163, 639)	686.0	1	3.26	0	0
	Venezuela (Bolivarian Republ	Americas	121 (71, 192)	994 (484, 1800)	1115.0	102040	3588.42	894	0.876127009
	Viet Nam	Western Pacific	574 (331, 941)	278 (130, 520)	852.0	1347	13.84	35	2.598366741
Excluded	Wallis and Futuna	Western Pacific				3	266.76	0	0
	Yemen	Eastern Mediterranean	52 (30, 86)	362 (167, 668)	414.0	2081	69.77	606	29.12061509
	Zambia	Africa	68 (40, 112)	612 (309, 1091)	680.0	17647	959.91	357	2.023006743
	Zimbabwe	Africa	5 (3, 7)	630 (312, 1230)	635.0	10034	675.1	277	2.760613913

*Based on eTable 3. Mean dietary saturated fat, omega-6 polyunsaturated fat, trans fat, cholesterol,
