# Supplementary material for: In Silico Study of Polyunsaturated Fatty Acids as Potential SARS-CoV-2 Spike Protein Closed Conformation Stabilizers: Epidemiological and Computational Approaches
Source: Molecules. 2021 Jan 29;26(3):711. doi: 10.3390/molecules26030711 (PMC7866518; doi:10.3390/molecules26030711)
Supplement: Supplementary file 1 [file molecules-26-00711-s001.zip › Table S2 (molecular docking results).pdf]

Table S2. Docking results of the fatty acid library

Common name	Fatty acid type	Name	Yasara structure (Kcal/mol)		Molegro Virtual Docker (score)	
			**Closed conformation**	**Open conformation**	**Closed conformation**	**Open conformation**
Tetracosahexaenoic acid (Nisinic acid)	24:6 (n-3)	*all-cis* -6,9,12,15,18,21-tetracosahexaenoic acid	9.43	4.78	-122.07	-77.47
Tetracosapentaenoic acid	24:5 (n-6)	*all-cis* -6,9,12,15,18-tetracosapentaenoic acid	8.58	4.54	-124.34	-97.01
Tetracosapentaenoic acid	24:5 (n-3)	*all-cis* -9,12,15,18,21-tetracosapentaenoic acid	8.98	4.56	-125.94	-94.46
Tetracosatetraenoic acid	24:4 (n-6)	*all-cis* -9,12,15,18-tetracosatetraenoic acid	8.40	4.46	-114.84	-83.79
Nervonic acid†	24:1 (n-9)	*cis* -15-tetracosenoic acid	7.91	4.41	-111.70	-76.45
Docosahexaenoic acid (DHA, Cervonic acid)	22:6 (n-3)	*all-cis* -4,7,10,13,16,19-docosahexaenoic acid	8.87	4.72	-117.85	-79.01
Docosapentaenoic acid (Osbond acid)	22:5 (n-6)	*all-cis* -4,7,10,13,16-docosapentaenoic acid	8.69	4.84	-103.09	-77.88
Docosapentaenoic acid (DPA, Clupanodonic acid)	22:5 (n-3)	*all-cis* -7,10,13,16,19-docosapentaenoic acid	8.51	4.73	-115.80	-88.64
Adrenic acid (AdA)	22:4 (n-6)	*all-cis* -7,10,13,16-docosatetraenoic acid	8.67	4.68	-121.12	-87.74
Docosadienoic acid	22:2 (n-6)	*all-cis* -13,16-docosadienoic acid	7.86	4.09	-108.34	-72.63
Erucic acid†	22:1 (n-9)	*cis* -13-docosenoic acid	7.72	4.49	-112.10	-80.60
Heneicosapentaenoic acid (HPA)	21:5 (n-3)	*all-cis* -6,9,12,15,18-heneicosapentaenoic acid	8.74	4.91	-117.33	-88.76
Bosseopentaenoic acid	20:5 (n-6)	5Z,8Z,10E,12E,14Z-eicosapentaenoic acid	8.59	4.78	-100.08	-78.25
Eicosapentaenoic acid (EPA, Timnodonic acid)	20:5 (n-3)	*all-cis* -5,8,11,14,17-eicosapentaenoic acid	8.58	5.56	-98.14	-77.58
Arachidonic acid (AA)	20:4 (n-6)	*all-cis* -5,8,11,14-eicosatetraenoic acid	8.24	4.80	-105.16	-70.68
Eicosatetraenoic acid (ETA)	20:4 (n-3)	*all-cis* -8,11,14,17-eicosatetraenoic acid	8.09	5.30	-107.78	-81.92
Mead acid	20:3 (n-9)	*all-cis* -5,8,11-eicosatrienoic acid	7.66	4.54	-107.58	-71.24
Dihomo-gamma-linolenic acid (DGLA)	20:3 (n-6)	*all-cis* -8,11,14-eicosatrienoic acid	8.23	4.46	-107.29	-70.14
sciadonic acid	20:3 (n-6)	(5Z,11Z,14Z)-eicosa-5,11,14-trienoic acid	7.99	4.68	-113.59	-80.21
Eicosatrienoic acid (ETE)	20:3 (n-3)	*all-cis* -11,14,17-eicosatrienoic acid	7.66	4.60	-113.03	-78.71
Eicosadienoic acid	20:2 (n-6)	*all-cis* -11,14-eicosadienoic acid	7.50	4.47	-103.16	-71.95
Eicosenoic acid gondoic	20:1 (n-9)	*cis* -11-eicosenoic acid	7.44	5.11	-105.12	-73.09
Stearidonic acid (SDA)	18:4 (n-3)	*all-cis* -6,9,12,15,-octadecatetraenoic acid	8.14	4.80	-97.99	-74.83
α-Parinaric acid	18:4 (n-3)	9E,11Z,13Z,15E-octadeca-9,11,13,15-tetraenoic acid	8.14	4.71	-103.78	-81.08
β-Parinaric acid	18:4 (n-3)	*all trans* -octadeca-9,11,13,15-tetraenoic acid	7.80	5.78	-107.30	-77.93
β-Calendic acid	18:3 (n-6)	8E,10E,12E-octadecatrienoic acid	8.19	5.41	-106.77	-71.35
Pinolenic acid	18:3 (n-6)	(5Z,9Z,12Z)-octadeca-5,9,12-trienoic acid	8.10	5.74	-104.43	-76.07
Gamma-linolenic acid (GLA)	18:3 (n-6)	*all-cis* -6,9,12-octadecatrienoic acid	7.59	4.71	-104.40	-82.38
α-Calendic acid	18:3 (n-6)	8E,10E,12Z-octadecatrienoic acid	7.46	5.53	-108.65	-75.28
Jacaric acid	18:3 (n-6)	8Z,10E,12Z-octadecatrienoic acid	7.45	4.53	-101.70	-83.14
Catalpic acid	18:3 (n-5)	9Z,11Z,13E-octadeca-9,11,13-trienoic acid	7.49	5.39	-106.06	-75.94
β-Eleostearic acid	18:3 (n-5)	9E,11E,13E-octadeca-9,11,13-trienoic acid	7.47	5.12	-107.11	-73.96
α-Eleostearic acid	18:3 (n-5)	9Z,11E,13E-octadeca-9,11,13-trienoic acid	7.45	4.61	-108.16	-86.64
Punicic acid	18:3 (n-5)	9Z,11E,13Z-octadeca-9,11,13-trienoic acid	7.44	4.69	-102.76	-76.17
Rumelenic acid	18:3 (n-3)	9E,11Z,15E-octadeca-9,11,15-trienoic acid	7.97	4.24	-104.27	-77.21
Alpha-linolenic acid (ALA)	18:3 (n-3)	*all-cis* -9,12,15-octadecatrienoic acid	7.50	4.54	-101.73	-79.05
Rumenic acid	18:2 (n-7)	9Z,11E-octadeca-9,11-dienoic acid	7.21	4.44	-102.88	-75.73
isorumenic acid	18:2 (n-6)	10E,12Z-octadeca-10,12-dienoic acid	7.27	4.42	-98.68	-82.21
Linoleic acid (LA)	18:2 (n-6)	*all-cis* -9,12-octadecadienoic acid	6.79	4.37	-104.76	-79.61
Oleic acid†	18:1 (n-9)	*cis* -9-octadecenoic acid	7.11	4.98	-105.92	-81.34
Hexadecatrienoic acid (HTA)	16:3 (n-3)	*all-cis* 7,10,13-hexadecatrienoic acid	7.69	5.25	-96.74	-81.09
Lignoceric acid	24:0	Tetracosanoic acid	8.02	4.10	-110.18	-72.26
Behenic acid	22:0	Docosanoic acid	7.37	4.09	-109.07	-72.02
Arachidic acid	20:0	Eicosanoic acid	6.59	4.19	-108.26	-73.12
Stearic acid	18:0	Octadecanoic acid	6.45	4.16	-103.98	-76.91
Palmitic acid	16:0	Hexadecanoic acid	7.16	4.94	-93.98	-70.38
